# Postintervention Dyspnea after Radiofrequency Catheter Ablation: Think of a Phrenic Nerve Injury

**DOI:** 10.1155/2017/6418070

**Published:** 2017-10-04

**Authors:** Liliana E. Ramos-Villalobos, Luis Colin Lizalde, Manlio F. Márquez, Pedro Iturralde, Francisco Castillo

**Affiliations:** ^1^Department of Electrophysiology, Instituto Nacional de Cardiología “Ignacio Chávez”, Mexico City, Mexico; ^2^Department of Cardiovascular Computer Tomography, Instituto Nacional de Cardiologia “Ignacio Chávez”, Mexico City, Mexico

## Abstract

Phrenic nerve injury (PNI) is a rare complication of catheter ablation therapy, most commonly observed in cryoablation of the right side pulmonary veins. We present a case of PNI after radiofrequency catheter ablation that developed acute dyspnea 24 hours after the intervention. Dyspnea is the main symptom of PNI, so the diagnosis should always be suspected if it appears after any type of catheter ablation involving the trajectory of the phrenic nerve. There is no specific treatment for PNI. The only maneuver that has been reported to accelerate the recovery of PNI is early stopping of the ablation therapy.

## 1. Introduction

Phrenic nerve injury (PNI) is a rare complication of atrial fibrillation (AF) ablation [1]. Its prevalence is higher with cryoablation (approximately 5%) than radiofrequency catheter ablation (RFCA; less than 1%) and therefore the development of diaphragmatic paralysis is monitored during the former procedure [2]. Although most cases of diaphragmatic paralysis show full recovery, it could be a potentially disabling complication [3]. We report a case of diaphragmatic paralysis secondary to PNI in the context of an uneventful RFCA of pulmonary veins, manifested as acute dyspnea 24 hours after the intervention.

## 2. Case Report

**Figure 1:**
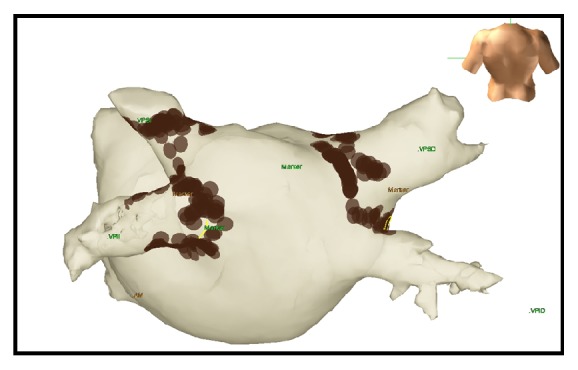
Electroanatomic map after atrial fibrillation ablation.

**Figure 2:**
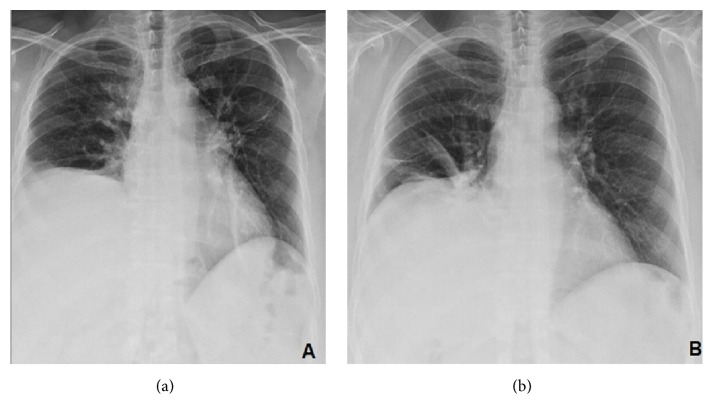
(a) Chest X-ray of the day after the radiofrequency catheter ablation (RFCA) in deep inspiration showing right hemidiaphragm paralyzed. (b) Chest X-ray 6 months after acute event.

**Figure 3:**
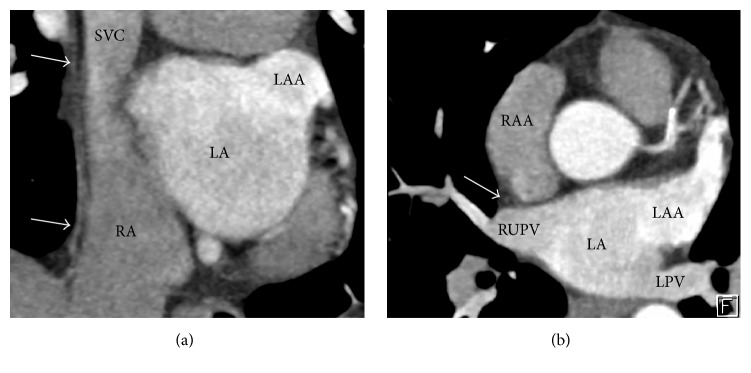
(a) Cardiac MDCT in paracoronal view showing the right pericardiophrenic bundle (arrows) adjacent to the superior vena cava (SVC) and right atrium (RA). (b) Paraxial view demonstrating the close relation (1 mm) of the pericardiophrenic bundle (arrow) to the right upper pulmonary vein (RUPV). RA: right atrium, LA: left atrium, LAA: left atrial appendage, RAA: right atrial appendage, and LPV: Left pulmonary veins.

A 51-year-old woman with diabetes mellitus, systemic arterial hypertension, bypass surgery, and obesity underwent RFCA of the pulmonary veins due to paroxysmal AF. Under conscious sedation, Lasso and Cordis 7-Fr catheters were used to map the left atrium and pulmonary veins (EnSite electroanatomic mapping system, St. Jude Medical, USA). Energy applied was limited to a power of 20 W achieving maximum temperature of 45°C. Radiofrequency applications, a total of 531 points giving 340 points to both right pulmonary veins, had an average length of 3 to 4 minutes per vein resulting in elimination of pulmonary vein action potentials (Figure 1). As routinely for RFCA, no phrenic nerve stimulation was performed during ablation of right pulmonary veins. During the procedure and immediate postoperative period the patient did not have any symptoms. The following day, on prone position and under routine supplementary oxygen she reported mild dyspnea that increased with sitting. Without supplementary oxygen, she developed oxygen desaturation of 85%. Pulmonary physical exploration showed absence of breathing sounds at the right base. Chest radiograph showed elevated right hemidiaphragm (Figure 2). ECG confirmed sinus rhythm and the echocardiogram was normal. The diagnosis of diaphragmatic dysfunction secondary to RFCA was made. The patient did not require noninvasive mechanical ventilation. She experienced complete recovery of symptoms with pulmonary physical therapy. Asymptomatic paralysis persisted in a 10-month follow-up (Figure 3).

## 3. Discussion

PNI in the context of RFCA has been reported in ablation of pulmonary veins (mostly right but also left) and ablation of superior vena cava (SVC) [4] and even in inappropriate sinus tachycardia [5]. The occurrence of PNI during RFCA for AF has been limited to case reports [6] and its prevalence in the context of RFCA for AF is low (0.48%) with a reported permanent damage of 0.17% [7]. It has been reported to occur more frequently in patients that underwent wide-area circumferential ablation whereas Yong reported an incidence of 30% [8]. The most common scenario in which PNI has been reported is cryoballoon ablation of the right-sided pulmonary veins. This has been observed in 8 to 11% of cryoablation procedures and was largely reversible. The mechanism of the occurrence of PNI is probably related to positioning the balloon further into the right pulmonary veins, as the phrenic nerve may run behind the right atrium. Such is the case as the undersizing of the balloon to a 23-mm versus a 28-mm balloon relative to the size of the vein that could contribute to this complication [9].

Three mechanisms might have been implicated in PNI: (1) direct heat transfer from the catheter contact site to the nerve, regardless of the energy source used for ablation; (2) effect of a high intensity electromagnetic field transiently generated at the catheter tip; (3) generation of a resonance current around the heart; furthermore, the PN is more sensitive to heating than the myocardium [3]. Sánchez-Quintana et al. described the close relationship between the atrial tissue surrounding the right superior pulmonary vein (RSPV) at its inferoanterior area, the RSPV, and the SVC with the phrenic nerve (Figure 3). Right Phrenic Nerve (RPN) descends vertically from its origin and continues along the right anterolateral surface SVC. Descending down the anterolateral wall of SVC, it turns posteriorly as it approaches the superior cavoatrial junction and follows in close proximity to the right-sided pulmonary veins. It is separated by only the pericardium at the anterolateral junction between the SVC and the right atrium. At this level, distance between the RSPV and RPN on an average is between 0 and 2.3 mm; in 6 specimens (32%) the RSPV is located <2 mm from RPN [10]. The closer relationship of the RPN to the RSPV makes it more susceptible to injury during ablation of the RSPV than during ablation of the Right Inferior Pulmonary Vein (RIPV). Ablation to achieve pulmonary vein isolation (PVI) has been highly successful in the management of AF. However, it is also associated with a variety of complications including PNI. The anatomic course of the phrenic nerve near the RSPV predisposes them to injury during the ablation.

Up to 31% of patients remain asymptomatic, but diaphragmatic dysfunction is an underdiagnosed cause of dyspnea and should always be considered as a differential diagnosis. Although dyspnea remains the cardinal symptom, the severity of it can vary depending on coexisting conditions such as obesity, weakness of other muscle groups, or underlying heart and lung disease, especially when they are supine [1]. Physical exploration reveals tachypnea, use of accessory muscles, and decreased diaphragmatic excursion can be detected by percussion of the lower rib cage at end expiration and end inspiration. The most characteristic physical sign is abdominal paradox, which is paradoxical inward motion of the abdomen as the rib cage expands during inspiration especially in supine position, and when this occurs in unilateral diaphragmatic paralysis, it suggests generalized weakness of the respiratory muscles. The diagnosis can be confirmed by a number of tests but the easiest one is the chest X-ray which shows elevated hemidiaphragm and basal subsegmental atelectasis [11]. Dyspnea could be resolved only with supplementary oxygen but, in some cases with severe respiratory distress, patients may require a few days of ventilatory support with noninvasive positive pressure ventilation or even invasive mechanical ventilation.

Early detection of PNI with phrenic nerve pacing is now the standard procedure during cryoablation in order to detect PNI earlier, to stop cryotherapy immediately, and to accelerate recovery of the injured nerve. Many strategies have been reported to be useful to avoid PNI in this setting. Some authors have postulated that preprocedural imaging could help to delineate the phrenic nerve course and therefore to avoid a PNI [12, 13]. Horton et al. [14]. reported a novel method of localization of the phrenic nerve with cardiac computed tomography and found that the imaged pericardiophrenic artery could reliably identify the approximate location of the RPN, suggesting that a phrenic nerve location within 10 mm of the RSPV poses a higher risk of PNI using balloon ablation devices. The improved temporal and spatial resolution allowed by multidetector computed tomography (MDCT) has facilitated the noninvasive assessment of cardiac anatomy before transcatheter electrophysiologic procedures. Clarification of spatial relations of phrenic nerves and key cardiac structures is important to decrease potential complications. The RPN was identified in 50 of 106 patients (47%). In the setting of electrophysiologic interventions, MDCT before a procedure may elucidate anatomic relationships and help minimize inadvertent complications [15]. In animals (rats) proximity of the ablation catheter to the phrenic nerve may be identified by capture during pacing [16]. Balloon inflation in the pericardial space has been described to protect the left phrenic nerve in the setting of epicardial ventricular tachycardia ablation. Nevertheless, techniques to protect the RPN during endocardial atrial ablation procedures have not been well described [17]. The likelihood of PNI associated with ablation using certain energy sources, the clinical course of this complication, and the possibility of recovery of PN function have not been reported [18]. The only parameter that predicts recovery has been the early interruption of the energy delivery. Short term outcomes from published cases are favorable with 81% of patients having a complete recovery after 6-7 months; however the recovery period can be as long as 28 months [1]. PNI has also been reported to occur with other energy sources for catheter ablation. It has been reported with radiofrequency hot balloon in 3.4% of cases and resolved in less than 3 months [19].

## 4. Conclusion

PNI is an uncommon complication of catheter ablation. It has been reported to be most commonly associated with atrial fibrillation ablation. It is important to consider PNI in the differential diagnosis of acute dyspnea after this intervention. Currently there is no reliable method to predict RPN injury prior to procedure. However, implementing vigorous monitoring of the nerve function can assure early detection and prevent permanent RPN. Because of this, it is very interesting for electrophysiologists and laboratory staffs to be familiar with and recognize this injury at the earliest to mitigate the damage.

## References

[1] Sacher F., Jais P., Stephenson K. (2007). Phrenic nerve injury after catheter ablation of atrial fibrillation. *Indian Pacing and Electrophysiology Journal*.

[2] Calkins H., Kuck K. H., Cappato R. (2012). 2012 HRS/EHRA/ECAS expert consensus statement on catheter and surgical ablation of atrial fibrillation: recommendations for patient selection, procedural techniques, patient management and follow-up, definitions, endpoints, and research trial design. *Journal of Interventional Cardiac Electrophysiology*.

[3] Durante-Mangoni E., Del Vecchio D., Ruggiero G. (2003). Right diaphragm paralysis following cardiac radiofrequency catheter ablation for inappropriate sinus tachycardia. *PACE - Pacing and Clinical Electrophysiology*.

[4] Miyazaki S., Ichihara N., Nakamura H. (2016). Prospective evaluation of electromyography-guided phrenic nerve monitoring during superior vena cava isolation to anticipate phrenic nerve injury. *Journal of Cardiovascular Electrophysiology*.

[5] Ibarra-Cortez S. H., Rodríguez-Mañero M., Kreidieh B., Schurmann P., Dave A. S., Valderrábano M. (2016). Strategies for phrenic nerve preservation during ablation of inappropriate sinus tachycardia. *Heart Rhythm*.

[6] Sacher F., Monahan K. H., Thomas S. P. (2006). Phrenic nerve injury after atrial fibrillation catheter ablation: characterization and outcome in a multicenter study. *Journal of the American College of Cardiology*.

[7] Cappato R., Calkins H., Chen S.-A. (2005). Worldwide survey on the methods, efficacy, and safety of catheter ablation for human atrial fibrillation. *Circulation*.

[8] Yong Ji S., Dewire J., Barcelon B. (2013). Phrenic nerve injury: An underrecognized and potentially preventable complication of pulmonary vein isolation using a wide-area circumferential ablation approach. *Journal of cardiovascular electrophysiology*.

[9] Packer D. L., Kowal R. C., Wheelan K. R. (2013). Cryoballoon ablation of pulmonary veins for paroxysmal atrial fibrillation: first results of the North American arctic front (STOP AF) pivotal trial. *Journal of the American College of Cardiology*.

[10] Sánchez-Quintana D., Cabrera J. A., Climent V., Farré J., Weiglein A., Ho S. Y. (2005). How close are the phrenic nerves to cardiac structures? Implications for cardiac interventionalists. *Journal of Cardiovascular Electrophysiology*.

[11] Gibson G. J. (1989). Diaphragmatic paresis: Pathophysiology, clinical features, and investigation. *Thorax*.

[12] Roka A., Heist E. K., Refaat M., Ruskin J., Mansour M. (2015). Novel technique to prevent phrenic nerve injury during pulmonary vein isolation using preprocedural imaging. *Journal of Cardiovascular Electrophysiology*.

[13] Kimura T., Takatsuki S., Fukumoto K. (2012). Electrical isolation of the superior vena cava using upstream phrenic pacing to avoid phrenic nerve injury. *PACE - Pacing and Clinical Electrophysiology*.

[14] Horton R., Di Biase L., Reddy V. (2010). Locating the right phrenic nerve by imaging the right pericardiophrenic artery with computerized tomographic angiography: Implications for balloon-based procedures. *Heart Rhythm*.

[15] Matsumoto Y., Krishnan S., Fowler S. J. (2007). Detection of phrenic nerves and their relation to cardiac anatomy using 64-slice multidetector computed tomography. *The American Journal of Cardiology*.

[16] Nichols N. L., Mitchell G. S. (2016). Quantitative assessment of integrated phrenic nerve activity. *Respiratory Physiology & Neurobiology*.

[17] Stark S., Roberts D. K., Tadros T., Longoria J., Krishnan S. C. (2017). Protecting the right phrenic nerve during catheter ablation: techniques and anatomical considerations. *HeartRhythm Case Reports*.

[18] Bai R., Patel D., Biase L. D. (2006). Phrenic nerve injury after catheter ablation: should we worry about this complication?. *Journal of Cardiovascular Electrophysiology*.

[19] Yamaguchi Y., Sohara H., Takeda H. (2015). Long-term results of radiofrequency hot balloon ablation in patients with paroxysmal atrial fibrillation: safety and rhythm outcomes. *Journal of Cardiovascular Electrophysiology*.

